# Molecular and Biochemical Impact of Selenium on the Acceleration of Ripening and Quality Changes in ‘Camarosa’ Strawberry Fruits

**DOI:** 10.3390/plants15121916

**Published:** 2026-06-21

**Authors:** Saeed Rezaei, Farhang Razavi, Leila Taghipour, Pedram Assar, Yolanda González-García, Antonio Juárez-Maldonado

**Affiliations:** 1 Department of Horticultural Sciences, Faculty of Agriculture, University of Zanjan, Zanjan 45371-38791, Iran; rezaei.s@znu.ac.ir; 2 Department of Horticultural Science, College of Agriculture, Jahrom University, Jahrom 74135-111, Iran; leilataghipour@jahromu.ac.ir (L.T.); pedramassar@jahromu.ac.ir (P.A.); 3 Campo Experimental Todos Santos, Centro de Investigación Regional Noroeste, Instituto Nacional de Investigaciones Forestales, Agrícolas y Pecuarias, La Paz 23070, Mexico; yolandaglezgar46@gmail.com; 4 Departamento de Botánica, Universidad Autónoma Agraria Antonio Narro, Saltillo 25315, Mexico

**Keywords:** antioxidant capacity, fruit physiology, gene expression, selenium, strawberry

## Abstract

Selenium is an essential micronutrient for humans, underscoring its importance in enhancing the nutritional and physiological attributes of agricultural and horticultural crops through exogenous application. At low doses, selenium improves growth and development, and increases crop yield and quality, particularly under stress conditions. It is believed that abscisic acid and sucrose work together to regulate strawberry (*Fragaria* × *ananassa* Duch.) fruit ripening. This study aimed to provide comprehensive biochemical and molecular insights into the selenium mediated effects on ripening and quality changes in ‘Camarosa’ strawberry fruits. Selenium treatment increased chlorophyll levels in leaves, suggesting a positive impact on overall plant health. Foliar application of 1 mM selenium significantly accelerated ripening. Treated fruits exhibited higher levels of total soluble solids, along with a decrease in titratable acidity. About lipid peroxidation indices, foliar application of 1 mM selenium decreases hydrogen peroxide and malondialdehyde. Consistently, flavonoids, phenolic compounds, anthocyanins, ascorbic acid, and antioxidant capacity, as well as the activity of the enzymes SOD, CAT, APX and PAL, were increased by selenium treatment. Interestingly, the ABA content in strawberry fruits also increased with selenium treatment. The selenium treatment upregulated genes involved in abscisic acid biosynthesis, phenolic compound biosynthesis, and anthocyanin production, namely, *FaNCED1*, *FaG2BD*, *FaCHS*, *FaPAL*, and *FaSUT1*. This study highlights the potential of selenium as a biostimulant and quality-enhancing agent in strawberries, improving fruit biochemical composition and ripening dynamics while contributing to better nutritional value and market appeal.

## 1. Introduction

Fruit ripening involves complex physiological and biochemical changes that enhance consumer appeal by affecting soluble solids, acidity, texture, and color [1]. Strawberry (*Fragaria* × *ananassa* Duch.) fruit, known for its sensory and nutritional properties, is rich in minerals, vitamins, and antioxidants such as phenolic acids, flavonoids, and anthocyanins [2,3]. The regulation of fruit ripening is a critical research area in fleshy fruit production, with strawberries providing insights into the roles of hormones and transcription factors in non-climacteric fruit development [4,5]. Understanding these mechanisms could enhance fruit quality through horticultural treatments.

Recent evidence highlights the crucial role of abscisic acid in strawberry ripening [6]. Manipulating genes such as *FaPYR1* and *FaNCED1*, which are involved in abscisic acid signaling and biosynthesis, respectively, has shown significant effects on fruit ripening [7]. Additionally, sucrose acts as a co-regulator with abscisic acid, influencing strawberry ripening and quality. Exogenous applications of abscisic acid and sucrose have been found to accelerate ripening and enhance quality indices beneficial for consumers [8,9].

Selenium is an essential micronutrient for humans, playing key roles in antioxidant defenses and thyroid hormone regulation [10]. However, its necessity for higher plants is uncertain, despite evidence of its beneficial effects. Selenium deficiency, affecting up to 1 billion people globally, leads to health issues such as oxidative stress and increased disease susceptibility [10]. Agricultural biofortification through fertilizers is proposed to enhance selenium bioavailability in the food supply, aiming to mitigate these deficiencies [11,12].

Selenium can be absorbed by plants in inorganic forms (selenate (SeO_4_^2−^) and selenite (SeO_3_^2−^)) or organic forms (selenium-amino acids). Selenate, being more soluble, is readily absorbed by plant roots [11]. Foliar application of selenium at low concentrations has been shown to increase selenium levels and improve growth and yield in various crops, including grapes [13,14]. Selenium enhances plant growth, development, and stress adaptation, with applications increasing antioxidant enzyme activity and reducing oxidative damage in plants under stress [15]. Fruit ripening, as observed in bananas, pears, peppers, and tomatoes, is considered a specialized form of senescence and involves increased reactive oxygen species levels due to declining antioxidant enzyme activities. Maintaining a balance between reactive oxygen species production and removal by antioxidant systems is crucial. Despite extensive research on the antioxidant capacity and phenolic content of strawberries, there remains limited information regarding the activities of major antioxidant enzymes during fruit development and ripening, particularly in relation to their response following selenium treatment [16].

Based on a pretest, this study aimed to provide biochemical and molecular insights into selenium-mediated effects on ripening and quality changes in strawberry fruit. This included characterizing changes in gene expression involved in abscisic acid, anthocyanin, and flavonoid biosynthesis, as well as sucrose accumulation. Additionally, the study evaluated the dynamics of abscisic acid, health-beneficial phytochemicals, and enzymatic antioxidant activities during the development and ripening of selenium-treated strawberry fruit. Finally, our research sought to offer insights into the potential and applied implications of selenium treatment for crop management and fruit quality enhancement, which are important for horticultural industries and horticulturists.

## 2. Results

### 2.1. Chlorophyll a and b Content of ‘Camarosa’ Strawberry Leaves

**Figure 1:**
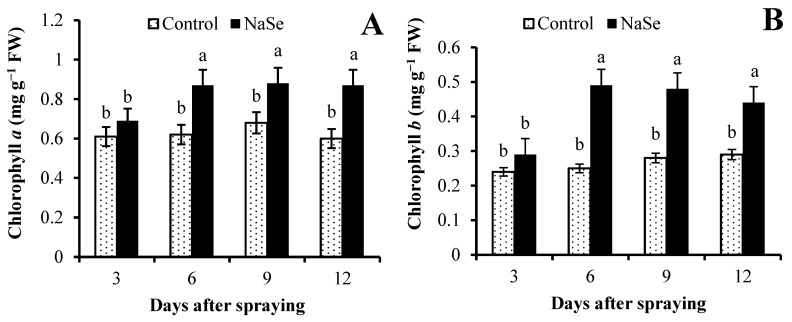
Chlorophyll *a* (**A**), and chlorophyll *b* (**B**) content of ‘Camarosa’ strawberry leaves following foliar application of 1 mM Na_2_SeO_4_. The data represent the interaction of time (days after spraying) and treatments (control and NaSe) and are the means of three replicates ± standard deviation. All statistical differences (by Duncan’s multiple range test, *p* ≤ 0.05) throughout the post-treatment period are shown in different letters.

The levels of chlorophyll *a* and *b* in selenium-treated plants were significantly higher compared to the control group at all sampling time points from the third to the twelfth day after treatment. The highest concentrations of chlorophyll *a* were observed in the selenium-treated samples on the sixth day after treatment and remained stable until the end of the experiment. In contrast, control plants exhibited a significant increase in chlorophyll *a* on the ninth day, followed by a significant decrease, ultimately reaching the lowest levels among all samples, similar to their initial values (Figure 1A). Chlorophyll *b* levels in the leaves of control plants increased as their fruits ripened, with significant differences emerging from the third sampling date onwards. We detected no differences between the first and second dates, nor between the third and fourth dates. Selenium-treated plants consistently exhibited significantly higher levels of chlorophyll *b* than the control group, with the highest levels observed on the sixth and ninth days after treatment (Figure 1B).

### 2.2. Titratable Acidity and Total Soluble Solid

**Figure 2:**
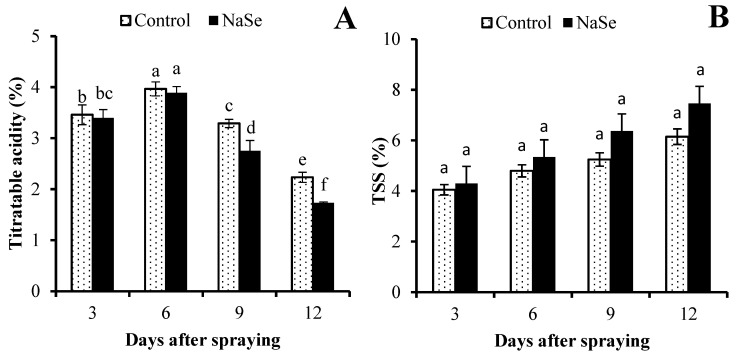
Titratable acidity (**A**), and Total soluble solid (**B**) of ‘Camarosa’ strawberry fruits following foliar application of 1 mM Na_2_SeO_4_. The data represent the interaction of time (days after spraying) and treatments (control and NaSe) and are the means of three replicates ± standard deviation. All statistical differences (by Duncan’s multiple range test, *p* ≤ 0.05) throughout the post-treatment period are shown in different letters.

The data indicate that selenium treatment influenced the titratable acidity, total soluble solid, and ascorbic acid content of ‘Camarosa’ strawberry fruits at various developmental stages. Both control and selenium-treated samples exhibited the highest titratable acidity on the sixth day. During the early stages (up to day 6), titratable acidity increased significantly in both treated and control groups, with no significant difference between them. However, in the later stages (days 6 to 12), a notable decline in titratable acidity was observed, particularly in selenium-treated fruits, which recorded the lowest levels on the twelfth day post-treatment (Figure 2A). Selenium application significantly enhanced the total soluble solid content in treated fruits compared to the control group, with the effect becoming more pronounced over time. On the twelfth day post-application, Se-treated fruits achieved the highest total soluble solid level, approximately 7.5%, while control fruits reached about 6.5% (Figure 2B).

### 2.3. Hydrogen Peroxide and Malondialdehyde

**Figure 3:**
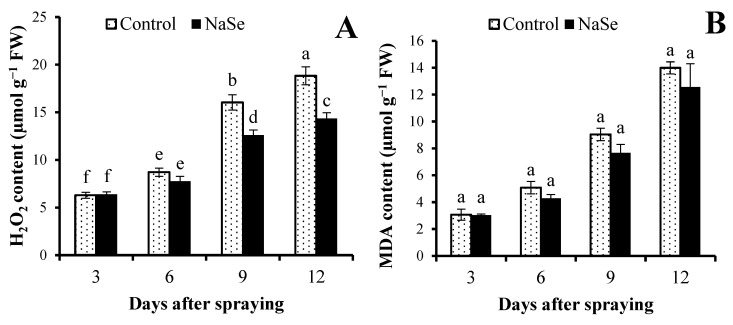
Hydrogen peroxide content (**A**), and Malondialdehyde content (**B**) of ‘Camarosa’ strawberry fruits following foliar application of 1 mM Na_2_SeO_4_. The data represent the interaction of time (days after spraying) and treatments (control and NaSe) and are the means of three replicates ± standard deviation. All statistical differences (by Duncan’s multiple range test, *p* ≤ 0.05) throughout the post-treatment period are shown in different letters.

The results depicted in Figure 3A indicate a consistent increase in hydrogen peroxide concentrations throughout the experimental period. No significant differences were observed between treated and control fruits until the midpoint of the sampling period. However, statistically significant differences emerged on days 9 and 12 post-treatment, with control fruits exhibiting higher hydrogen peroxide content than treated fruits. Similarly, Figure 3B demonstrates a parallel trend in malondialdehyde levels between treated and control fruits, with a statistically significant divergence observed starting on the sixth day after foliar application, mirroring the trend observed for hydrogen peroxide levels.

### 2.4. Total Flavonoid Content, Total Phenol Content, Total Anthocyanin Content, Ascorbic Acid, 2,2-diphenyl-1-picrylhydrazyl Scavenging Capacity, and Enzyme Activity

**Figure 4:**
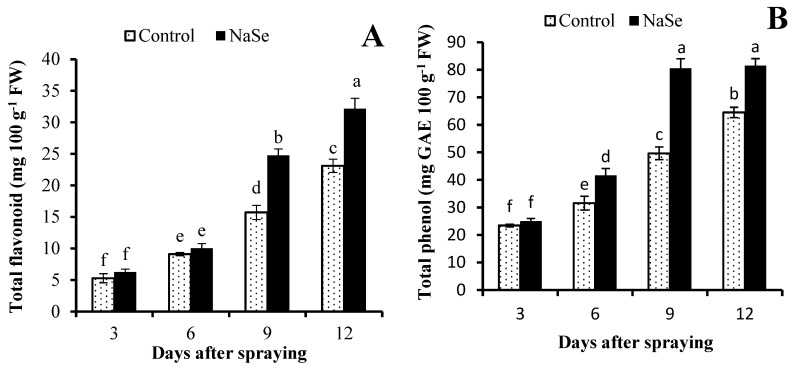

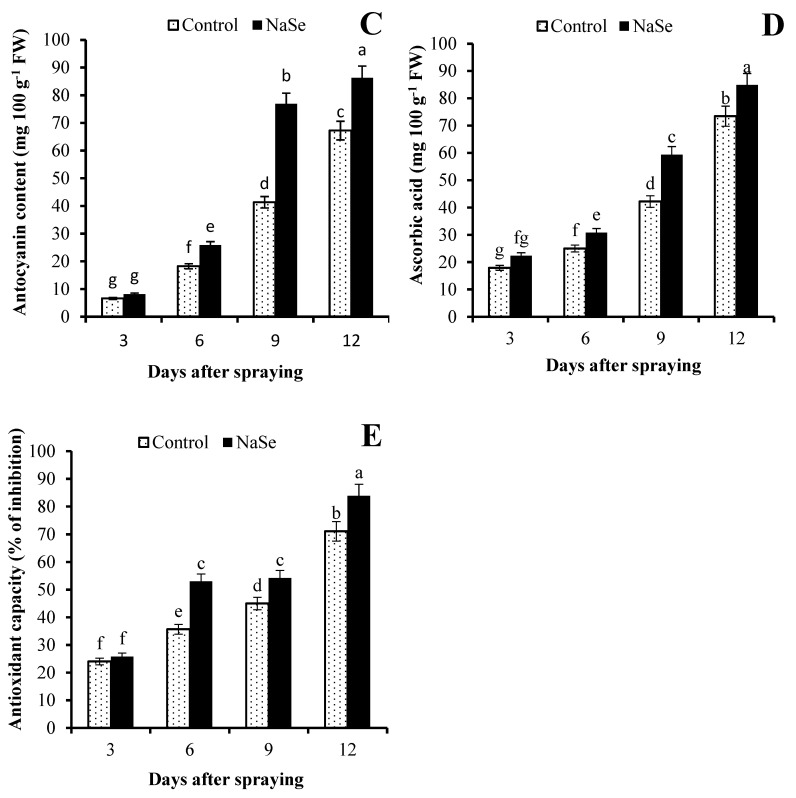
Total flavonoid (**A**), Total phenol (**B**), Anthocyanin content (**C**), Ascorbic Acid (**D**), and 2,2-diphenyl-1-picrylhydrazyl scavenging capacity (**E**) of ‘Camarosa’ strawberry fruits following foliar application of 1 mM Na_2_SeO_4_. The data represent the interaction of time (days after spraying) and treatments (control and NaSe) and are the means of three replicates ± standard deviation. All statistical differences (by Duncan’s multiple range test, *p* ≤ 0.05) throughout the post-treatment period are shown in different letters.

The results demonstrated a significant increase in the flavonoid content of whole fruits in both the control and treated groups throughout the experiment period. In the second half of the period, selenium-treated fruits consistently exhibited higher levels of total flavonoids compared to the control group. Consequently, the selenium-treated fruits had the highest amount of total flavonoid by the end of the experiment (Figure 4A).

For the first three days following selenium foliar treatment, the total phenol values in both treated and control fruits were similar. Subsequently, the total phenol content increased significantly in both groups. In the control group, this increase persisted until the end of the experiment, whereas in the treated group, it continued until the ninth day post-treatment. During the final three days of the experiment, the total phenol levels in the treated fruits remained constant. Throughout the period of increasing total phenol levels, the treated fruits consistently exhibited significantly higher total phenol levels compared to the control fruits (Figure 4B).

The data revealed that during the post-treatment period, the anthocyanin content of the fruits increased progressively. Treated fruits consistently exhibited higher anthocyanin content compared to the control fruits, with differences between the two groups becoming significant starting on the sixth day and persisting until the end of the experiment (Figure 4C).

Ascorbic acid concentration increased steadily during fruit ripening, while selenium-treated fruits consistently had higher ascorbic acid levels throughout the study period. The difference between selenium-treated fruits and the control group became statistically more evident in the second half of the sampling period. Treated fruits had the highest concentration of ascorbic acid on the twelfth day after treatment (Figure 4D).

At the first sampling point, the 2,2-diphenyl-1-picrylhydrazyl scavenging capacities of the control and treated fruits were identical. However, over time and continuing until the end of the experiment, both groups exhibited a continuous and significant increase in antioxidant capacity. During all three subsequent sampling times, the treated fruits consistently demonstrated higher antioxidant capacity compared to the control fruits (Figure 4E).

**Figure 5:**
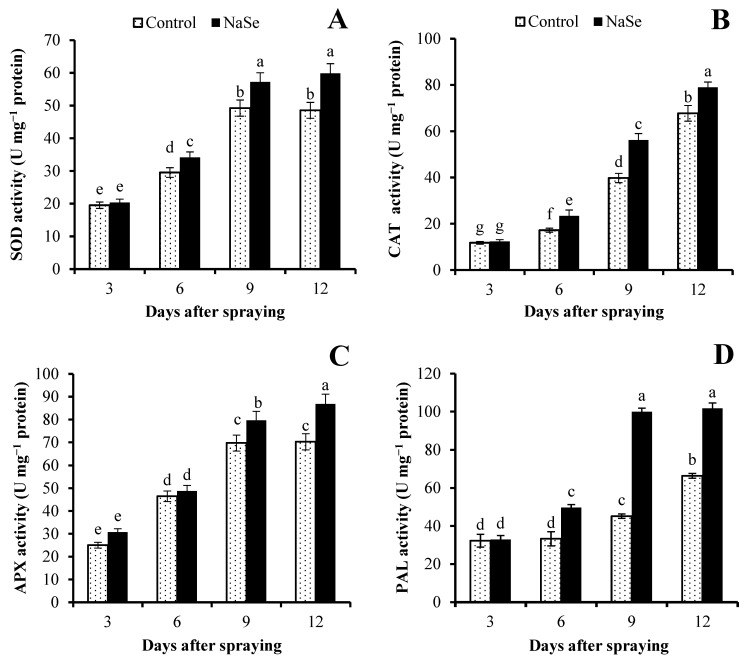
Superoxide dismutase activity (**A**), Catalase activity (**B**), Ascorbate peroxidase activity (**C**) and Phenylalanine ammonia-lyase (**D**) of ‘Camarosa’ strawberry fruits following foliar application of 1 mM Na_2_SeO_4_. The data represent the interaction of time (days after spraying) and treatments (control and NaSe) and are the means of three replicates ± standard deviation. All statistical differences (by Duncan’s multiple range test, *p* ≤ 0.05) throughout the post-treatment period are shown in different letters.

The investigation of changes in enzymatic antioxidant activity revealed that the activity levels of superoxide dismutase, catalase, and ascorbate peroxidase increased over time during the post-treatment period. Treated fruits consistently exhibited higher antioxidant activity compared to control fruits (Figure 5). The differences in the activity levels of superoxide dismutase and catalase between the treated and control groups became significant from the sixth day, while the difference in ascorbate peroxidase activity became significant from the ninth day and continued until the end of the experiment (Figure 5A–C).

Results indicated that phenylalanine ammonia-lyase enzyme activity in the control fruits remained stable until the midpoint of the sampling period, after which it increased. The selenium-treated fruits, on the other hand, showed a significant and progressive increase in phenylalanine ammonia-lyase activity until the ninth day after treatment. The difference in phenylalanine ammonia-lyase activity between the control and treated fruits became significant on the sixth day after treatment and persisted until the end of the experiment. The enzyme activity in the treated fruits peaked in the latter half of the experiment, significantly surpassing that of the control fruits (Figure 5D).

### 2.5. Abscisic Acid Content, and the Expression of Key Genes Involved in the Regulation of Fruit Ripening

**Figure 6:**
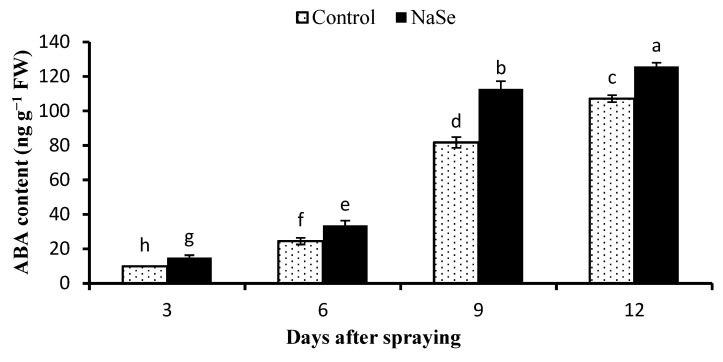
Abscisic acid content in ‘Camarosa’ strawberry fruits following foliar application of 1 mM Na_2_SeO_4_. The data represent the interaction of time (days after spraying) and treatments (control and NaSe) and are the means of three replicates ± standard deviation. All statistical differences (by Duncan’s multiple range test, *p* ≤ 0.05) throughout the post-treatment period are indicated by different letters.

The data indicate that abscisic acid content in the fruits increased over time during the post-treatment period, mirroring the pattern observed for anthocyanin content. Throughout the sampling period, treated fruits consistently displayed higher abscisic acid levels compared to the control fruits. The differences between the treated and control groups became significant on the sixth day and persisted until the end of the experiment period (Figure 6).

**Figure 7:**
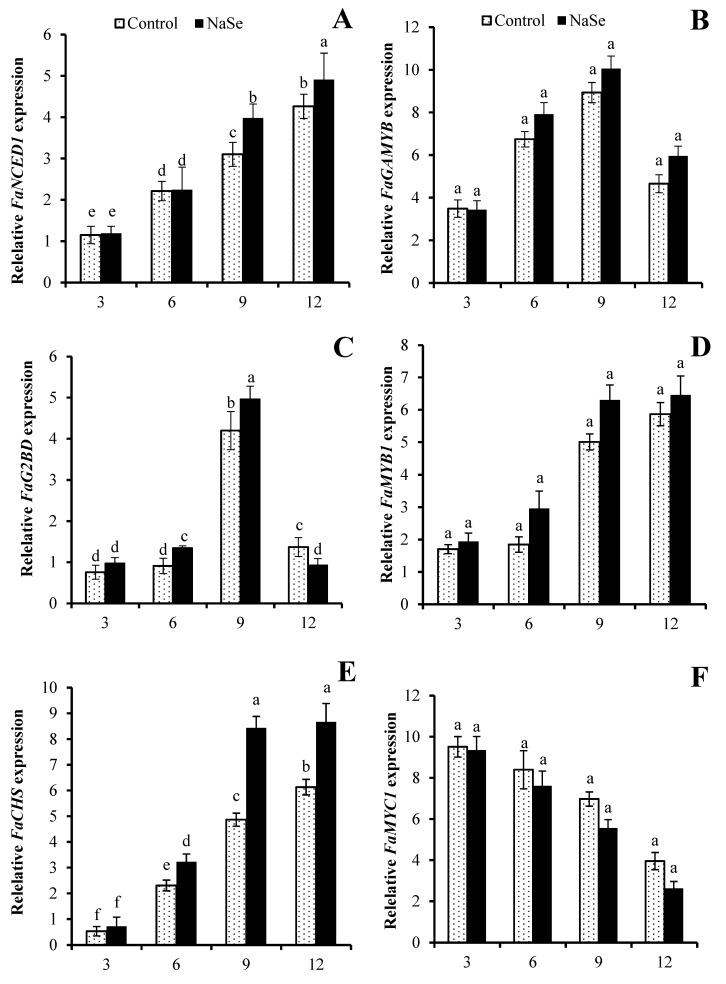

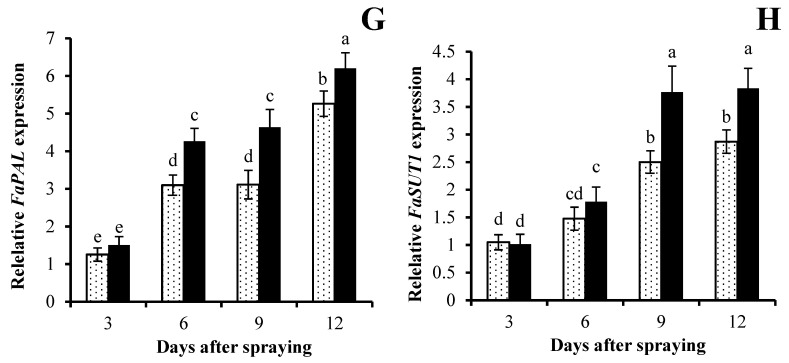
Relative expression of *FaNCED1* (**A**), *FaGAMYB* (**B**), *FaG2BD* (**C**), *FaMYB1* (**D**) *FaCHS* (**E**), *FaMYC1* (**F**), *FaPAL* (**G**), and *FaSUT1* (**H**) genes in ‘Camarosa’ strawberry fruits following foliar application of 1 mM Na_2_SeO_4_. The data represent the interaction of time (days after spraying) and treatments (control and NaSe) and are the means of three replicates ± standard deviation. All statistical differences (by Duncan’s multiple range test, *p* ≤ 0.05) throughout the post-treatment period are indicated by different letters.

Figure 7 examines changes in the expression levels of key genes involved in the regulation of abscisic acid biosynthesis and ripening (*FaNCED1*, *FaGAMYB*), anthocyanin synthesis pathway (*FaMYB1*, *FaCHS*, *FaMYC1*, *FaPAL*), and sucrose transport and accumulation (*FaSUT1*).

The results revealed that in both control and treated fruit groups, the expression levels of the *FaNCED1*, *FaGAMYB*, *FaG2BD*, *FaMYB1*, *FaCHS*, *FaPAL*, and *FaSUT1* genes increased throughout the post-treatment period until its conclusion, with higher expression levels observed in the treated fruits compared to the controls (Table S3). *FaMYC1* was the only gene that decreased over time (Figure 7F). Furthermore, the control group exhibited higher expression levels than the Na_2_SeO_4_-treated plants (Table S3).

## 3. Discussion

### 3.1. Impact of Selenium on of Photosynthetic Pigments

The results obtained here, regarding the increase in chlorophyll *a* and *b* content under selenium treatment are consistent with various reports (Figure 1, Table S1). Several studies have demonstrated that the application of low concentrations of selenium significantly enhances various physiological traits in plants, resulting in improved growth and performance [17,18]. Researchers have demonstrated that selenium, at low concentrations, promotes the biosynthesis of photosynthetic pigments by safeguarding chloroplast enzymes and facilitating pigment production [19]. Suggested that the increase in chlorophyll content may result from selenium’s ability to inhibit reactive oxygen species. Furthermore, selenium’s antioxidant properties protect plant cell membranes from lipid peroxidation [20]. Dong et al. [21] reported that optimal selenium application significantly elevated the levels of chlorophyll *a*, chlorophyll *b*, and carotenoids in *Lycium chinense*. Other studies reported increased chlorophyll levels in Se-treated tomatoes [22].

### 3.2. Impact of Selenium on Physicochemical Parameters of Strawberry Fruits

Titratable acidity and total soluble solid are crucial factors influencing strawberry quality [23]. Total soluble solid serves as a critical quality indicator strongly linked to fruit structure and ripening processes. Multiple factors contribute to the increase in total soluble solid content throughout ripening, such as heightened respiration rates, increased activity of cell wall-degrading enzymes, accelerated polysaccharide decomposition, and a more concentrated fruit extract [24]. Sucrose, a prevalent carbohydrate derived from photosynthesis, is transported to the fruit, enhancing total soluble solid and the proportion of soluble sugars. Additionally, the increased activity of the enzyme sucrose phosphate synthase facilitates sucrose production in fruit [25]. Numerous studies on horticultural crops have demonstrated that appropriate selenium concentrations can significantly enhance total soluble solid content in produce. For instance, selenium boosted photosynthesis in passion fruit, which thereby increased sugar accumulation and significantly impacted total soluble solid content [26]. Furthermore, selenium foliar application has been linked to increased grape berry growth, sugar accumulation, total soluble solid content, and overall berry quality [13,14]. Similarly, a positive relationship between selenium concentration and total soluble solid has been found in peach [27] and pomegranate [28]. Selenium enrichment in strawberry fruits has also been associated with elevated soluble solids content [29]. All of these findings highlight selenium’s potential in enhancing sugar metabolism and accelerating fruit ripening, thereby improving produce quality.

Researchers proposed that selenium foliar application can enhance concentrations of soluble sugars, soluble solids, and vitamin C while simultaneously reducing organic acid levels, ultimately improving grape quality. This metabolic shift may contribute to the observed decrease in total acid content and the increase in soluble solids during fruit ripening (Figure 2, Table S1). Notably, research on pears involving selenium application demonstrated similar results [30], with decreased titratable acidity and increased total soluble solid further supporting the outcomes of our study.

### 3.3. Stress Biomarkers: Hydrogen Peroxide and Malondialdehyde

Reactive oxygen species comprise highly reactive molecules, including free radicals such as superoxide (O_2_^•−^) and hydroxyl (OH^•^), as well as non-radical species like (hydrogen peroxide) and singlet oxygen (^1^O_2_). These are byproducts of normal metabolic processes in plants and can accumulate excessively under different stresses [31]. Excess reactive oxygen species can deteriorate cell organelles and membrane components, disrupt protein synthesis, ion transport, and enzyme activity, and lead to physiological or programmed cell death, affecting overall plant health [32]. In chloroplasts, reactive oxygen species accumulation disrupts photosynthetic electron transport and photosystem II assembly, impairing photosynthetic efficiency and plant growth. This results in abnormal growth, accelerated senescence, and premature aging of plant tissues, ultimately impacting yield and quality [32]. Malondialdehyde is the primary byproduct and marker of the oxidation of unsaturated fatty acids (membrane peroxidation). Its content, along with the content of hydrogen peroxide, serves as critical indicators for assessing oxidative stress in plants [23].

As observed here, the production of hydrogen peroxide and malondialdehyde increased over time. However, applying selenium significantly decreased the content of both compounds in strawberries (Figure 3, Table S1). This behavior has been previously reported by several authors and is due to the activation of the antioxidant system [33,34,35].

### 3.4. Impact of Selenium on Antioxidant System and Secondary Metabolites

Selenium influences metabolic pathways by modulating the cellular redox balance and synthesizing antioxidants aimed at reducing reactive oxygen species levels [35]. Selenium application has been reported to enhance plant antioxidant capacity both directly and indirectly, preserve cell membrane integrity, and delay senescence by reducing malondialdehyde and hydrogen peroxide levels through both enzymatic and non-enzymatic mechanisms [33]. Studies on horticultural crops (Pezzarossa et al. [34] have shown that selenium supplementation, either through soil or foliar treatments, can improve antioxidant capacity or reduce oxidative stress, thereby enhancing plant performance and maintaining fruit quality during its development and ripening stages. In one study, treatments with 2.5 and 5 mg L^−1^ of selenium led to a decrease in the accumulation of malondialdehyde and hydrogen peroxide in quinoa (*Chenopodium quinoa*) plants [36]. Moreover, selenium treatment has been shown to decrease hydrogen peroxide content in strawberries during the postharvest period [23]. The findings of this study, indicating lower levels of malondialdehyde and hydrogen peroxide (Figure 3, Table S1), and higher flavonoid, phenol, ascorbic acid, antioxidant capacity and antioxidant enzymatic activities in selenium-treated fruits during the post-treatment period (Figure 4 and Figure 5, Table S2), are consistent with the abovementioned reports.

The relationships between selenium supplementation, phenylalanine ammonia-lyase enzyme activity, total phenol content, total flavonoid content, anthocyanin content, and antioxidant capacity in fruits are well-supported by existing literature. Selenium is known to enhance the activity of antioxidant enzymes, such as glutathione peroxidase, which helps mitigate oxidative stress and stimulate the production of secondary metabolites, including phenolic compounds [37]. Phenylalanine ammonia-lyase is a key enzyme in the phenylpropanoid pathway, using phenylalanine as a substrate amino acid for the biosynthesis of a wide range of phenolic compounds [38]. A study examined the relationship between phenylalanine ammonia-lyase activity and flavonoid accumulation in different strawberry cultivars, finding that higher phenylalanine ammonia-lyase activity was associated with increased levels of various flavonoid compounds in the fruits, including anthocyanins, flavonols, and proanthocyanidins [39].

It has been reported that selenium at low doses increased the leaf phenolic content of hydroponically grown tomato plants. Also, spraying tomato plants with selenate led to selenium biofortified fruits that had higher amounts of the antioxidant flavonoids naringenin, chalcone, and kaempferol [10]. The results obtained here on strawberry fruit are consistent with previous reports, indicating that exogenous Se application can modulate phenylalanine ammonia-lyase activity to stimulate the biosynthesis of phenolic and flavonoid compounds, thereby enhancing the antioxidant capacity and overall fruit quality (Figure 4, Table S2).

### 3.5. Impact of Selenium on the Expression of Key Genes Involved in the Regulation of Fruit Ripening

Interestingly, the ripening of strawberries depends on the accumulation of abscisic acid, as observed here (Figure 6), which is regulated by the FaNCED1 gene. This is true even though strawberries are not climacteric fruits. Along with the accumulation of ABA, the production of anthocyanins (the pigment that gives ripe fruit its red color) is induced, as well as the accumulation of sucrose, which provides sweetness [40]. Jia et al. [7] reported that abscisic acid is a key regulator of fruit development, playing a crucial role in the regulation of fruit ripening and the expression of genes related to the accumulation of beneficial phytochemicals in strawberries. They mentioned that during strawberry fruit development, abscisic acid levels increase gradually, peaking at the onset of ripening. This increase in abscisic acid triggers the expression of genes involved in fruit softening, chlorophyll breakdown, anthocyanin and ascorbic acid biosynthesis, and volatile compound production. In another study Zahedi et al. [3] reported an increase in the phytohormone abscisic acid in strawberry leaves in response to foliar spray with selenium nanoparticles on plants grown on non-saline and different saline soils. Mattus-Araya et al. [9] treated detached immature fruits of *Fragaria chiloensis* with exogenous abscisic acid and stored them at 20 °C. The treated fruits turned color faster than the control group. This was accompanied by higher levels of anthocyanin accumulation and higher transcript levels of genes involved in the phenylpropanoid/flavonoid and anthocyanin pathways, specifically *FcPAL*, *FcCHS*, and *FcANS*. They concluded that abscisic acid induced transcriptional modifications led to accelerate fruit ripening and color development in this non-climacteric fruit.

The results obtained here demonstrate this behavior. The *FaNCED1*, *FaGAMYB*, *FaG2BD*, *FaMYB1*, *FaCHS*, *FaPAL*, and *FaSUT1* genes were overexpressed as the fruit matured over time (Figure 7). Moreover, the results showed that selenium treatment increased the expression levels of most of these genes in the fruits of the treated plants compared to the control plants (Table S3). Studies have shown that selenium upregulates the expression of phenylalanine ammonia-lyase encoding genes, enhancing phenylalanine ammonia-lyase activity and promoting the synthesis of phenolic compounds, thereby improving fruit quality and organ antioxidant properties. For instance, the upregulation of phenylalanine ammonia-lyase in selenium-treated *Brassica napus* plants revealed selenium’s role in regulating transcriptional networks responsible for producing phenylpropanoid derivatives, including phenols and flavonoids, which act as antioxidants to scavenge oxidative damage [41]. Chen et al. [42] reported that selenium treatment enhanced photosynthesis and up-regulated sucrose transporter gene expression. This led to more sucrose synthesis and accumulation, which intensified the expression level of anthocyanin biosynthesis genes in the hypocotyl of radish sprouts.

In detail, *FaNCED1* (*Fragaria* × *ananassa* 9-Cis-Epoxycarotenoid Dioxygenase) encodes the enzyme NCED, a key regulator of abscisic acid biosynthesis. The *FaGAMYB* (*Fragaria* × *ananassa* Gibberellic Acid-Regulated MYB) encodes an R2R3-MYB transcription factor, which positively regulates genes involved in the biosynthesis of proanthocyanidins and anthocyanins in strawberry fruits [43]. The *FaMYB1* (*Fragaria* × *ananassa* MYB) gene was also found to have synergistic effects in determining the red color of strawberry fruit through the regulation of flavonoid biosynthesis in response to increased endogenous abscisic acid during the final stages of fruit development [44]. The *FaMYC1* (*Fragaria* × *ananassa* MYC) gene encodes a bHLH (basic Helix-Loop-Helix) transcription factor, found to interact with *FaMYB1* to activate anthocyanin biosynthesis genes [43]. The *FaCHS* (*Fragaria* × *ananassa* Chalcone Synthase) encodes chalcone synthase, the first committed step in the flavonoid biosynthesis pathway [45]. The *FaPAL* (*Fragaria* × *ananassa* Phenylalanine Ammonia-Lyase) encodes phenylalanine ammonia-lyase, a key enzyme in the phenylpropanoid pathway, catalyzing the conversion of phenylalanine to trans-cinnamic acid, a precursor for various phenolic compounds [44].

The *FaSUT1* (*Fragaria* × *ananassa* Sucrose Transporter) plays a vital role in strawberry fruit development and sugar accumulation. Its function is critical for sucrose translocation, a precursor for various secondary metabolites, including phenolic compounds, with a specific role as a signal in strawberry ripening. Jia et al. [7] highlighted the significant role of sucrose in strawberry fruit growth and development, identifying it as a crucial regulatory signal for fruit ripening. Their research showed that exogenous sucrose application induced an increase in abscisic acid accumulation, significantly accelerating fruit ripening. They identified *FaSUT1* as a key component responsible for sucrose accumulation during fruit development. Silencing *FaSUT1* through RNA interference resulted in reduced sucrose and abscisic acid levels, thereby inhibiting fruit ripening. Conversely, overexpressing *FaSUT1* led to higher sucrose and abscisic acid levels, which accelerated the ripening process. Additionally, Luo et al. (2019) [8] reported that abscisic acid and sucrose jointly influence strawberry fruit ripening. Their findings showed that endogenous levels of abscisic acid and sucrose, along with the expression of related signaling and ripening genes such as *NCED1*, *NCED2* (abscisic acid biosynthesis regulators), *SnRK2.2* (an abscisic acid signaling regulator), *SuSy* (sucrose synthase gene), *MYB5*, *CEL1*, and *CEL2* (genes related to pigment and cell-wall metabolism), were significantly elevated by treatments with abscisic acid or sucrose, and even more so with a combined abscisic acid and sucrose treatment. In a recent study, it was demonstrated that nano-selenium treatment could mitigate the negative effects of fungicides on strawberry flavor and antioxidant capacity by modulating abscisic acid biosynthesis and ripening-related transcription factors [46]. Their results showed that treated plants exhibited better fruit quality, evidenced by higher levels of volatiles, anthocyanins, enzyme activities, and 2,2-diphenyl-1-picrylhydrazyl scavenging ability, along with lower reactive oxygen species levels compared to control plants. These improvements were linked to the regulation of genes such as *FaRIF*, *FaSnRK1*, *FaMYB10*, *FaMYB1*, *FaSnRK2.6*, and *FaABI1*, which are involved in flavor and ripening processes.

## 4. Materials and Methods

### 4.1. Plant Material, Growing Conditions, and Treatments

**Figure 8:**
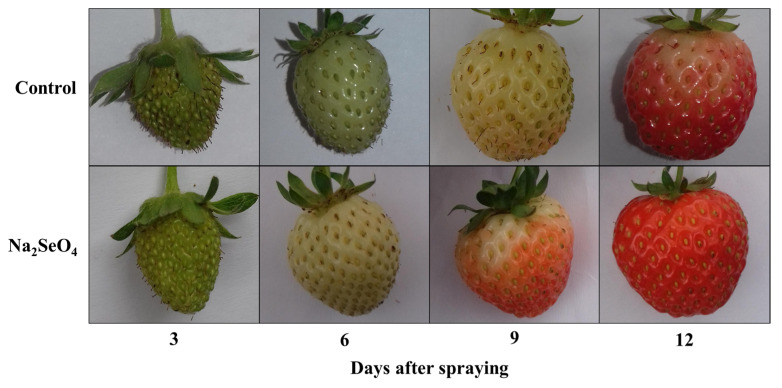
Strawberry fruits collected at four sampling times after treatment application.

The experiment was conducted in a greenhouse near Zanjan city (Zanjan province, Iran), located at an altitude of 1602 m above sea level, with coordinates 48°39′ longitude and 36°71′ latitude. The greenhouse maintained an average relative humidity of 50–60% and a maximum/minimum temperature of 20/25 ± 2 °C. The plant material used was ‘Camarosa’ strawberry plants at their full growth stage, free of diseases. They were grown in a perlite medium as a soilless culture. Regular pest and fungal disease management, weeding, and removal of old or diseased leaves were performed as necessary. The growth and developmental progression of strawberry fruit were classified into four distinct stages-green, white, turning, and red-as previously characterized [4]. To standardize the experiment, fruits at the green stage, were maintained, and any remaining flowers and fruits were meticulously removed. The experiment was conducted using a split-plot factorial design (main plot was sampling time at four levels, and the subplot was a foliar spray of Na_2_SeO_4_ at two levels) with three replications (four plants per experimental unit). A foliar spray of Sodium selenate (Na_2_SeO_4_) with 95% purity (Sigma Aldrich, St. Louis, MI, USA) was applied evenly at a concentration of 1 mM, using 40 mL per plant during the evening hours. For the control group, each plant received 40 mL of distilled water by foliar spray. Fruit and leaf samples were collected at four sampling times: three, six, nine, and twelve days after treatment (Figure 8). The harvested samples were immediately frozen in liquid nitrogen and stored in aluminum foil at −80 °C until further analysis.

A pretest similar to the main experiment was conducted to determine the optimal non-toxic concentration of Na_2_SeO_4_. Various concentrations, ranging from zero to 2 mM, were individually applied by foliar spray. Physicochemical indices, such as total soluble solids and fruit firmness fruit were evaluated (Figure S1). Additionally, color and stress indicators like leaf discoloration, necrosis, wilting, curling, and signs of toxicity or deficiency, were visually evaluated at the sampling times. The results revealed that 1 mM Na_2_SeO_4_ was the optimal dosage. Although there were no statistically significant differences between the treatments, this concentration exhibited the highest TSS and firmness values. Furthermore, no toxicity was observed in any treatment.

### 4.2. Chlorophyll a and b

Chlorophyll *a* and *b* in strawberry leaves were measured following the method outlined [47]. Approximately 1.0 g of leaf tissue was homogenized in a mortar with 10 mL of 80% acetone. The homogenate was centrifuged at 5000 rpm for 10 min. The filtrate was then subjected to UV-VIS spectrophotometric analysis, with absorbance readings taken at 645 nm and 663 nm. Chlorophyll *a* and *b* concentrations were calculated using the following equations and expressed in milligrams per gram of fresh weight:

(1) $$\mathrm{Chlorophyll}\;a\;=\;(12.25\;\times \;A_{663}\;-\;2.79\;\times \;A_{645})$$

Chlorophyll *b* = (21.50 × *A*_645_ − 5.10 × *A*_663_) (2)

### 4.3. Titratable Acidity, Total Soluble Solid, and Ascorbic Acid Content

Titratable acidity was measured following AOAC standard methods and expressed as a percentage [48]. Total soluble solid was assessed using a refractometer (ARBO-45, manufactured in Tokyo, Japan) and reported in Brix degrees. The quantification of ascorbic acid levels followed the method described by Liguori et al. [49]. Ten grams of the blended fresh strawberry sample were taken, 100 mL of metaphosphoric acid (HPO_3_) was added, and the mixture was then filtered through Whatman No. 1 filter paper. A 10 mL aliquot of the filtered solution was titrated with 2,6-dichlorophenol-indophenol reagent until a persistent pink coloration appeared, lasting for 15 s. The ascorbic acid content was expressed as milligrams per 100 g of fresh weight.

### 4.4. Hydrogen Peroxide and Malondialdehyde Content

Fresh strawberry samples were homogenized in ice-cold 50 mM potassium phosphate buffer (pH 7.0) containing 1% polyvinylpolypyrrolidone. The homogenate was centrifuged for 15 s, and the supernatant was used for malondialdehyde and hydrogen peroxide content determination. Hydrogen peroxide was measured using the titanium sulfate method, where the absorbance of the titanium-peroxide complex was recorded at 410 nm [50]. Malondialdehyde, a biomarker of lipid peroxidation, was quantified using the thiobarbituric acid reactive substances assay, with the pink malondialdehyde-thiobarbituric acid adduct detected at 532 nm [51]. The concentrations of malondialdehyde and hydrogen peroxide were expressed as micromoles per gram of fresh weight of strawberry fruit.

### 4.5. Antioxidant Enzymes Extraction and Activity Assay

Strawberry fruit tissues (2 g) were homogenized in 20 mL of 50 mM sodium phosphate buffer (pH 7.8) containing 1.0 mM EDTA, 0.3% Triton X-100, and 1% polyvinyl polypyrrolidone 3 min. The homogenate was then filtered through cheesecloth and then centrifuged at 15,000 rpm for 20 min at 4 °C. The resulting supernatant was collected as the crude extract for antioxidant enzyme assays. The method of Mousavizadeh-Fashian et al. [52] was used to determine the activity of antioxidant enzymes, including superoxide dismutase, catalase, and ascorbate peroxidase. The activities of superoxide dismutase, catalase, and ascorbate peroxidase were expressed as units per milligram protein. Bradford method [53] was applied to measure the protein content using bovine serum albumin as a standard.

### 4.6. Phenylalanine Ammonia-Lyase Enzyme Activity, Total Phenol Content, Total Flavonoid Content, Total Anthocyanin Content, and 2,2-diphenyl-1-picrylhydrazyl Scavenging Capacity

Phenylalanine ammonia-lyase enzyme assay was performed following the method of Sardari et al. [54]. One gram of fruit samples was homogenized in 3 mL of ice-cold 0.1 M sodium borate buffer (pH 7.0) containing 1.4 mM 2-mercaptoethanol and 0.1 g of insoluble polyvinylpyrrolidone. The homogenate was filtered through cheesecloth, and the filtrate was centrifuged at 16,000 rpm for 15 min at 4 °C. The supernatant served as the enzyme source. The phenylalanine ammonia-lyase activity was determined by measuring the conversion rate of L-phenylalanine to trans-cinnamic acid. A reaction mixture containing 0.4 mL of enzyme extract, 0.5 mL of 0.1 M borate buffer (pH 8.8), and 0.5 mL of 12 mM L-phenylalanine in the same buffer was incubated for 30 min at 30 °C. The optical density was measured at 290 nm, and the amount of trans-cinnamic acid formed was calculated using its extinction coefficient of 9630 M^−1^. One unit of phenylalanine ammonia-lyase activity was defined as the amount of the enzyme that was required to form 1 nmol of trans-cinnamic acid per min and the data were expressed as units per milligram of protein.

Phenolic compounds in strawberries were extracted and quantified following a previously reported method [55]. Two grams of the sample were mixed with 5 mL of acidified methanol (0.1% *v*/*v* HCl), and the mixture was sonicated for 30 min at 20 ± 2 °C in an ultrasonic bath. After sonication, the mixture was centrifuged at 10,000 rpm for 10 min, and the supernatant was collected. The pellet was re-extracted once more in the same manner, and both supernatants were combined. This extract was used to quantify the total phenolic content using the Folin–Ciocalteu assay with minimal modifications. A 0.5 mL sample extract was mixed with 2.5 mL of 0.2 N Folin–Ciocalteu reagent, followed by the addition of 2 mL of 7.5% aqueous sodium carbonate solution. The mixture was incubated at room temperature in the dark for 2 h, and then the absorbance was measured at 760 nm. A calibration curve was prepared using gallic acid as a standard compound, and the results were expressed on a fresh weight basis as milligrams of gallic acid equivalents per 100 g of fresh weight. Total flavonoid content in the extracted sample was measured using a colorimetric assay [55]. Initially, 0.3 mL of 5% NaNO_2_ was added to a 15 mL test tube containing 4 mL of distilled water and 1 mL of the sample. The mixture was vortexed and allowed to sit for 5 min at room temperature. Then, 0.3 mL of 10% AlCl_3_ was added, vortexed again, and left to sit for 6 min at room temperature. Following this, 2.4 mL of distilled water was added to 2 mL of 1 N NaOH, vortexed, and the final volume was brought to 10 mL. The absorbance was measured at 510 nm. Catechin was used as the standard, and the results were expressed as milligrams of catechin equivalent per 100 g of fresh weight.

Total anthocyanin content in fruit juice was determined using the pH differential method [56]. Absorbance was measured at 510 and 700 nm in buffers at pH 1.0 and 4.5. The following formula was used to calculate anthocyanin content:

(3) $$A\;=\;[(A_{510}\;-\;A_{700})\;pH\;1.0\;-\;(A_{510}\;-\;A_{700})\;pH\;4.5$$

A molar extinction coefficient of 22,400 for pelargonidin 3-glucoside was used for strawberry fruit juice. Results were expressed as milligrams of Pg3G equivalents per 100 g of fresh weight. The antioxidant capacity was determined following 2,2-diphenyl-1-picrylhydrazyl (DPPH) free radical scavenging capacity for which 70 µL of the centrifuged extract was mixed with 1930 µL of the DPPH solution with a concentration of 0.1 mM. The reaction mixture was shaken fast and kept in the dark at room temperature for 30 min. The absorbance of the solution was measured at 517 nm, and the 2,2-diphenyl-1-picrylhydrazyl scavenging capacity was calculated using the following formula:

(4) $$DPPH\;scavenging\;capacity\;(\%)\;=\;[1\;-\;(A_{0}/A_{1})]\;\times 100$$

where *A*_0_ is the absorption of the sample and *A*_1_ is the absorption of the blank 2,2-diphenyl-1-picrylhydrazyl solution [57].

### 4.7. Abscisic Acid Content

For the analysis of endogenous abscisic acid content, 2 g of frozen fruit powder were homogenized with 70% (*v*/*v*) methanol and left to stir overnight at 4 °C, following the method described by Kelen et al. [58]. The resulting extract was filtered through a Whatman No. 1 filter, and methanol was evaporated under vacuum. The aqueous phase was adjusted to pH 8.5 using 0.1 M phosphate buffer and partitioned with ethyl acetate three times. After removing the ethyl acetate phase, the pH of the aqueous phase was lowered to 2.5 using 1 N HCl. This solution was then partitioned with diethyl ether and passed through anhydrous sodium sulfate. The diethyl ether phase was evaporated under vacuum to yield a dry residue containing hormones. The dry residue was dissolved in 2 mL of methanol for abscisic acid analysis. High-performance liquid chromatography (HPLC) was employed for the analysis, using a Knuer 2050 system equipped with a C18 column (150 × 4.6 mm). The flow rate was set at 0.8 mL min^−1^, and each analysis utilized an injection volume of 20 μL. The endogenous abscisic acid content was monitored at 265 nm and expressed as nanograms per gram based on fresh weight.

### 4.8. Expression of Key Genes Involved in the Regulation of Abscisic Acid Biosynthesis, Phenolic Compounds Biosynthesis, and Sucrose Transport

Primers for strawberry genes were designed using data from the NCBI database, utilizing AlleleID 7.8 software to target exons and junctions of genes related to fruit ripening in strawberries (Table S4). These primers were obtained from Macrogen, South Korea, and diluted to a concentration of 100 pmol using DEPC-treated water (Macrogen, Seoul, South Korea). Total RNA was extracted from fruit tissue samples using RiboEX (GeneAll, Seoul, South Korea), following the protocols of Chomczynski and Sacchi [59]. The quantity of RNA was measured using a NanoDrop 2000 Spectrophotometer (Thermo Scientific, Waltham, MA, USA), which provided the RNA concentration in nanograms per microliter (ng µL^−1^). RNA quality was assessed by 1% agarose gel electrophoresis in TAE buffer, with 10 μL of RNA and 2 μL of loading dye buffer loaded into the wells. Electrophoresis was conducted at 80 volts for 30–50 min [60]. cDNA was synthesized using the HyperScript cDNA synthesis kit (GeneAll, South Korea) according to the manufacturer’s instructions, with a minimum of 1μ of RNA. The reaction mixture (Table 1) was incubated at 55 °C for 60 min, followed by 95 °C for 5 min. The synthesized cDNA was stored at −80 °C [61]. qPCR was performed using a Rotor-Gene 6000 (QIAGEN, Venlo, Netherlands) with the 5x EvaGreen master mix (Solis BioDyne, Tartu, Estonia). The qPCR reaction components and thermal cycling conditions are listed in Table 2 and Table 3. Negative control samples, where cDNA was replaced with deionized water, were included in each run [62]. A housekeeping gene, interspacer 26S–18S, which shows stable and constitutive expression during strawberry fruit development and under various experimental conditions, was selected for normalization of qPCR data. The stability of the interspacer 26S–18S gene was validated under our experimental conditions prior to gene expression analysis. Relative expression levels of target genes were calculated using the 2^−ΔΔCt^ method after normalization to this reference gene.

### 4.9. Experimental Design and Statistical Analysis

The experiment was conducted using a split-plot factorial design with three replications (four plants per experimental unit). The main plot was sampling time at four levels (three, six, nine, and twelve days after treatment), and the subplot was a foliar spray of Na_2_SeO_4_ at two levels (0 µM (control) and 1 mM). When statistical differences were observed, means were compared using Duncan’s multiple range test at a 5% probability level. Data analysis was performed using SPSS software (version 22). qPCR data were analyzed using GenEX software (Multid, Sweden) based on the method of Pfaffl [63]. Charts and tables were created using Microsoft Office Excel and Word 2016 software.

## 5. Conclusions

This study provides molecular and biochemical insights into how selenium affects the ripening and quality of strawberries, which can be used as a model to investigate non-climacteric fruits. Findings revealed that foliar application of selenium influences the expression of genes involved in abscisic acid and phenolic compound biosynthesis and sucrose accumulation. Considering the importance of selenium supplementation for human health, this approach improves not only fruit ripening and quality indices, but also helps meet daily dietary selenium requirements. Thus, selenium foliar application emerges as a promising strategy to optimize the nutritional value and health benefits of strawberries, a highly popular superfruit.

## Figures and Tables

**Table 1:** Materials for cDNA synthesis.

Material	Volume (μL)
Total RNA	5
Oligo	1
DEPC-treated water	5
RT Master	10

**Table 2:** qPCR materials.

Material	Volume (μL)
5x EvaGreen qPCR mix	4
Forward primer	1
Reverse primer	1
Template cDNA	3
Nuclease-free water	11

**Table 3:** qPCR thermal program.

Stage	Temperature (°C)	Time	Number of Cycles
Initial Denaturation	95	15 min	1
Denaturation	95	20 s	40–45
Annealing	52–60	20 s	40–45
Extension	55	20 s	40–45
Melt Curve	99	-	1

## Data Availability

Data will be made available on request.

## Supplementary Materials

The following supporting information can be downloaded at: https://www.mdpi.com/article/10.3390/plants15121916/s1, Supplementary material. Figure S1. Total Soluble Solid (A) and Firmness (B) of ‘Camarosa’ strawberry fruits following foliar application of Na_2_SeO_4_. Table S1. Effect of the main factors of the variables: Chlorophyll *a*, Chlorophyll *b*, Titratable acidity, Total soluble solid, Hydrogen peroxide, and Malondialdehyde. Table S2. Effect of the main factors of the variables: Total flavonoid, Total phenol, Anthocyanin, Ascorbic Acid, DPPH, SOD, CAT, APX, PAL. Table S3. Effect of the main factors of the variables: ABA, *FaNCED1*, *FaGAMYB*, *FaMYB1*, *FaMYC1*, *FaCHS*, *FaPAL*, and *FaSUT1*. Table S4. Primer’s information.

## References

[1] Jia H., Wang Y., Sun M., Li B., Han Y., Zhao Y., Li X., Ding N., Li C., Ji W. (2013). Sucrose functions as a signal involved in the regulation of strawberry fruit development and ripening. New Phytol..

[2] Aharoni A., Ric De Vos C.H., Wein M., Sun Z., Greco R., Kroon A., Mol J.N.M., O’Connell A.P. (2001). The strawberry FaMYB1 transcription factor suppresses anthocyanin and flavonol accumulation in transgenic tobacco. Plant J..

[3] Zahedi S.M., Abdelrahman M., Hosseini M.S., Hoveizeh N.F., Tran L.S.P. (2019). Alleviation of the effect of salinity on growth and yield of strawberry by foliar spray of selenium-nanoparticles. Environ. Pollut..

[4] Vallarino J.G., Osorio S., Bombarely A., Casañal A., Cruz-Rus E., Sánchez-Sevilla J.F., Amaya I., Giavalisco P., Fernie A.R., Botella M.A. (2015). Central role of FaGAMYB in the transition of the strawberry receptacle from development to ripening. New Phytol..

[5] Chen R., Mao L., Guan W., Wei X., Huang Z., Wu Y. (2022). ABA-mediated miR5290 promotes anthocyanin biosynthesis by inhibiting the expression of FaMADS1 in postharvest strawberry fruit. Postharvest Biol. Technol..

[6] Chai Y.M., Jia H.F., Li C.L., Dong Q.H., Shen Y.Y. (2011). FaPYR1 is involved in strawberry fruit ripening. J. Exp. Bot..

[7] Jia H.F., Chai Y.M., Li C.L., Lu D., Luo J.J., Qin L., Shen Y.Y. (2011). Abscisic acid plays an important role in the regulation of strawberry fruit ripening. Plant Physiol..

[8] Luo Y., Ge C., Ling Y., Mo F., Yang M., Jiang L., Chen Q., Lin Y., Sun B., Zhang Y. (2019). ABA and sucrose co-regulate strawberry fruit ripening and show inhibition of glycolysis. Mol. Genet. Genom..

[9] Mattus-Araya E., Guajardo J., Herrera R., Moya-León M.A. (2022). ABA speeds up the progress of color in developing *F. chiloensis* fruit through the activation of PAL, CHS and ANS, key genes of the phenylpropanoid/flavonoid and anthocyanin pathways. Int. J. Mol. Sci..

[10] Schiavon M., dall’Acqua S., Mietto A., Pilon-Smits E.A.H., Sambo P., Masi A., Mario M. (2013). Selenium fertilization alters the chemical composition and antioxidant constituents of tomato (*Solanum lycopersicon* L.). J. Agric. Food Chem..

[11] Sanchez-Rodas D., Mellano F., Martínez F., Palencia P., Giraldez I., Morales E. (2016). Speciation analysis of Se-enriched strawberries (*Fragaria ananassa* Duch) cultivated on hydroponics by HPLC-TR-HG-AFS. Microchem. J..

[12] Santiago F.E.M., Silva M.L.D.S., Ribeiro F., Cipriano P.E., Guilherme L.R.G. (2018). Influence of sulfur on selenium absorption in strawberry. Acta Sci. Agron..

[13] Zhu S., Liang Y., An X., Kong F., Gao D., Yin H. (2017). Changes in sugar content and related enzyme activities in table grape (*Vitis vinifera* L.) in response to foliar selenium fertilizer. J. Sci. Food Agric..

[14] Zhu S., Liang Y., Gao D., An X., Kong F. (2017). Spraying foliar selenium fertilizer on quality of table grape (*Vitis vinifera* L.) from different source varieties. Sci. Hortic..

[15] Liu X.W., Wang Q.L., Duan B.H., Lin Y.M., Zhao X.H., Hu C.X., Zhao Z.Q. (2015). Effects of selenite addition on selenium absorption, root morphology and physiological characteristics of rape seedlings. J. Appl. Ecol..

[16] López A.P., Gochicoa M.T.N., Franco A.R. (2010). Activities of antioxidant enzymes during strawberry fruit development and ripening. Biol. Plant.

[17] Ahmad R., Waraich E.A., Nawaz F., Ashraf M.Y., Khalid M. (2016). Selenium (Se) improves drought tolerance in crop plants: A myth or fact?. J. Sci. Food Agric..

[18] Pennanen A., Xue T., Hartikainen H. (2002). Protective role of selenium in plant subjected to severe UV irradiation stress. J. Appl. Bot. Food Qual..

[19] Hawrylak-Nowak B. (2009). Beneficial effects of exogenous selenium in cucumber seedlings subjected to salt stress. Biol. Trace Elem. Res..

[20] Sun H.W., Ha J., Liang S.X., Kang W.J. (2010). Protective role of selenium on garlic growth under cadmium stress. Soil. Sci. Plant Anal..

[21] Dong J.Z., Wang Y., Wang S.H., Yin L.P., Xu G.J., Zheng C., Lei C., Zhang M.Z. (2015). Selenium increases chlorogenic acid, chlorophyll and carotenoids of *Lycium chinense* leaves. J. Sci. Food Agric..

[22] Mozafariyan M., Pessarakli M., Saghafi K. (2015). Effects of selenium on some morphological and physiological traits of tomato plants grown under hydroponic condition. J. Plant Nutr..

[23] Lin Y., Liang W., Cao S., Tang R., Mao Z., Lan G., Zhou S., Zhang Y., Li M., Wang Y. (2023). Postharvest application of sodium selenite maintains fruit quality and improves the gray mold resistance of strawberry. Agronomy.

[24] Vargas R.C., Defilippi B.G., Valdes G.H., Robledo M.P., Prieto E.H. (2008). Effect of harvest time and L-cysteine as an antioxidant on flesh browning of fresh-cut cherimoya (*Annona cherimola* Mill). Agric. Res..

[25] Tavarini S., Innocenti E.D., Remorini D., Massai R., Guidi L. (2008). Antioxidant capacity, ascorbic acid, total phenols and carotenoids changes during harvest and after storage of Hayward kiwifruit. Food Chem..

[26] Huang W., Shen M., Liu Z., Zhang Y., Wang D. (2020). Effects of different application methods and application rates of biological nano-selenium on the quality of passion fruit. Med. Plant.

[27] Zhang H., Han T., Tian L., Wang Y.N., Jia H.J. (2010). Accumulation of Se in peach, jujube and strawberry after spraying Se fertilizer on leaves. J. Fruit. Sci..

[28] Zahedi S.M., Hosseini M.S., Meybodi N., Silva J. (2019). Foliar application of selenium and nano-selenium affects pomegranate (*Punica granatum* cv. Malase Saveh) fruit yield and quality. S. Afr. J. Bot..

[29] Zhang Z.Y., Gao S., Shan C.J. (2020). Effects of sodium selenite on the antioxidant capacity and the fruit yield and quality of strawberry under cadmium stress. Sci. Hortic..

[30] Yuan C., Bu H., Zhao J., Liu J., Wang G., Yuan H., Wang A. (2023). Effect of Se application on selenium accumulation and fruit quality in pear (*Pyrus ussuriensis*). Acta Physiol. Plant..

[31] Gill S.S., Tuteja N. (2010). Reactive oxygen species and antioxidant machinery in abiotic stress tolerance in crop plants. Plant Physiol. Biochem..

[32] Sahu P.K., Jayalakshmi K., Tilgam J., Gupta A., Nagaraju Y., Kumar A., Hamid S., Singh H.V., Minkina T., Rajput V.D. (2022). ROS generated from biotic stress: Effects on plants and alleviation by endophytic microbes. Front. Plant Sci..

[33] Hartikainen H., Xue T., Piironen V. (2000). Selenium as an anti-oxidant and pro-oxidant in ryegrass. Plant Soil..

[34] Pezzarossa B., Remorini D., Gentile M.L., Massai R. (2012). Effects of foliar and fruit addition of sodium selenate on selenium accumulation and fruit quality. J. Sci. Food Agric..

[35] Feng R., Wei C., Tu S. (2013). The roles of selenium in protecting plants against abiotic stresses. Environ. Exp..

[36] Khalofah A., Migdadi H., El-Harty E. (2021). Antioxidant enzymatic activities and growth response of quinoa (*Chenopodium quinoa* Willd) to exogenous selenium application. Plants J..

[37] Jing D.W., Du Z.Y., Ma H.L., Ma B.Y., Liu F.C., Song Y.G., Li L. (2017). Selenium enrichment, fruit quality and yield of winter jujube as affected by addition of sodium selenite. Sci. Hortic..

[38] Cheynier V., Comte G., Davies K.M., Lattanzio V., Martens S. (2013). Plant phenolics: Recent advances on their biosynthesis, genetics, and ecophysiology. Plant Physiol. Biochem..

[39] Carbone F., Preuss A., De Vos R.C.H., D’amico E., Perrotta G., Bovy A.G., Martens S., Rosati C. (2009). Developmental, genetic, and environmental factors affect the expression of flavonoid genes, enzymes, and metabolites in strawberry fruits. Plant Cell Environ..

[40] Li B.-J., Grierson D., Shi Y., Chen K.-S. (2022). Roles of abscisic acid in regulating ripening and quality of strawberry, a model non-climacteric fruit. Hortic. Res..

[41] Ulhassan Z., Gill R.A., Ali S., Mwamba T.M., Ali B., Wang J., Huang Q., Aziz R., Zhou W., Dual T. (2019). Behavior of selenium: Insights into physio-biochemical, anatomical and molecular analyses of four *Brassica napus* cultivars. Chemosphere.

[42] Chen J., Chen H., Wang H., Zhan J., Yuan X., Cui J., Su N. (2023). Selenium treatment promotes anthocyanin accumulation in radish sprouts (*Raphanus sativus* L.) by its regulation of photosynthesis and sucrose transport. Food Res. Int..

[43] Liu J., Wang J., Wang M., Zhao J., Zheng Y., Zhang T., Xue L., Lei J. (2021). Genome-wide analysis of the R2R3-MYB gene family in *Fragaria* × *ananassa* and its function identification during anthocyanins biosynthesis in pink-flowered strawberry. Front. Plant Sci..

[44] Wang H., Zhang H., Yang Y., Li M., Zhang Y., Liu J., Dong J., Li J., Butelli E., Xue Z. (2020). The control of red colour by a family of MYB transcription factors in octoploid strawberry (*Fragaria* × *ananassa*) fruits. Plant Biotechnol. J..

[45] Sun W., Meng X., Liang L., Jiang W., Huang Y., He J., Hu H., Almqvist J., Gao X., Wang L. (2015). Molecular and biochemical analysis of chalcone synthase from Freesia hybrid in flavonoid biosynthetic pathway. PLoS ONE.

[46] Liu Y., Liu R., Li F., Yu S., Nie Y., Li J.Q., Pan C., Zhu W., Zhou Z., Diao J. (2024). Nano-selenium repaired the damage caused by fungicides on strawberry flavor quality and antioxidant capacity by regulating ABA biosynthesis and ripening-related transcription factors. Pestic. Biochem. Physiol..

[47] Lichtenthaler H.K., Buschmann C. (2001). Chlorophylls and carotenoids: Measurement and characterization by UV-VIS spectroscopy. Curr. Protoc. Food Anal. Chem..

[48] Latimer G.W., AOAC International (2012). Official Methods of Analysis of AOAC International.

[49] Liguori G., Gaglio R., Settanni L., Inglese P., D’Anna F., Miceli A. (2021). Effect of Opuntia ficus-indica mucilage edible coating in combination with ascorbic acid, on strawberry fruit quality during cold storage. J. Food Qual..

[50] Velikova V., Yordanov I., Edreva A. (2000). Oxidative stress and some antioxidant systems in acid rain-treated bean plants: Protective role of exogenous polyamines. Plant Sci..

[51] Heath R.L., Packer L. (1968). Photoperoxidation in isolated chloroplasts. I. Kinetics and stoichiometry of fatty acid peroxidation. Arch. Biochem. Biophys..

[52] Mousavizadeh-Fashian A., Rahimi M.M., Hosseinifarahi M. (2024). Alleviating the adverse effects of drought stress on physiological characters of filed pumpkin by salicylic acid and selenium. Russ. J. Plant Physiol..

[53] Bradford M.M. (1976). A rapid and sensitive method for the quantitation of microgram quantities of protein utilizing the principle of protein-dye binding. Anal. Biochem..

[54] Sardari M., Rezayian M., Niknam V. (2022). Comparative study for the effect of selenium and nano-selenium on wheat plants grown under drought stress. Russ. J. Plant Physiol..

[55] Hwang H., Kim Y.J., Shin Y. (2019). Influence of ripening stage and cultivar on physicochemical properties, sugar and organic acid profiles, and antioxidant compositions of strawberries. Food Sci. Biotechnol..

[56] Ayala-Zavala J., Wang S.Y., Wang C.Y., González-Aguilar G.A. (2004). Effect of storage temperatures on antioxidant capacity and aroma compounds in strawberry fruit. LWT.

[57] Brand-Williams W., Cuvelier M.E., Berset C. (1968). Use of a free radical method to evaluate antioxidant activity. LWT.

[58] Kelen M., Demiralay E.C., Şen S., Alsancak G.Ö. (2004). Separation of abscisic acid, indole-3-acetic acid, gibberellic acid in 99 R (*Vitis berlandieri* x *Vitis rupestris*) and rose oil (*Rosa damascena* Mill.) by reversed phase liquid chromatography. Turk. J. Chem..

[59] Chomczynski P., Sacchi N. (2006). The single-step method of RNA isolation by acid guanidinium thiocyanate-phenol-chloroform extraction: Twenty-something years on. Nat. Protoc..

[60] Rio D.C., Ares M.J., Hannon G.J., Nilsen T.W. (2010). Purification of RNA using TRIzol (TRI reagent). Cold Spring Harb. Protoc..

[61] Schmittgen T.D., Livak K.J. (2008). Analyzing real-time PCR data by the comparative CT method. Nat. Protoc..

[62] Bustin S.A., Benes V., Garson J.A., Hellemans J., Huggett J., Kubista M., Mueller R., Nolan T., Pfaffl M.W., Shipley G.L. (2009). The MIQE guidelines: Minimum information for publication of quantitative real-time PCR experiments. Clin. Chem..

[63] Pfaffl M.W.A. (2001). New mathematical model for relative quantification in real-time RT-PCR. Nucleic Acids Res..

