# Supplementary material for: Molecular and Biochemical Impact of Selenium on the Acceleration of Ripening and Quality Changes in ‘Camarosa’ Strawberry Fruits
Source: Plants (Basel). 2026 Jun 21;15(12):1916. doi: 10.3390/plants15121916 (PMC13306251; doi:10.3390/plants15121916)
Supplement: Supplementary file 1 [file plants-15-01916-s001.zip › plants-4352217-supplementary.pdf]

A

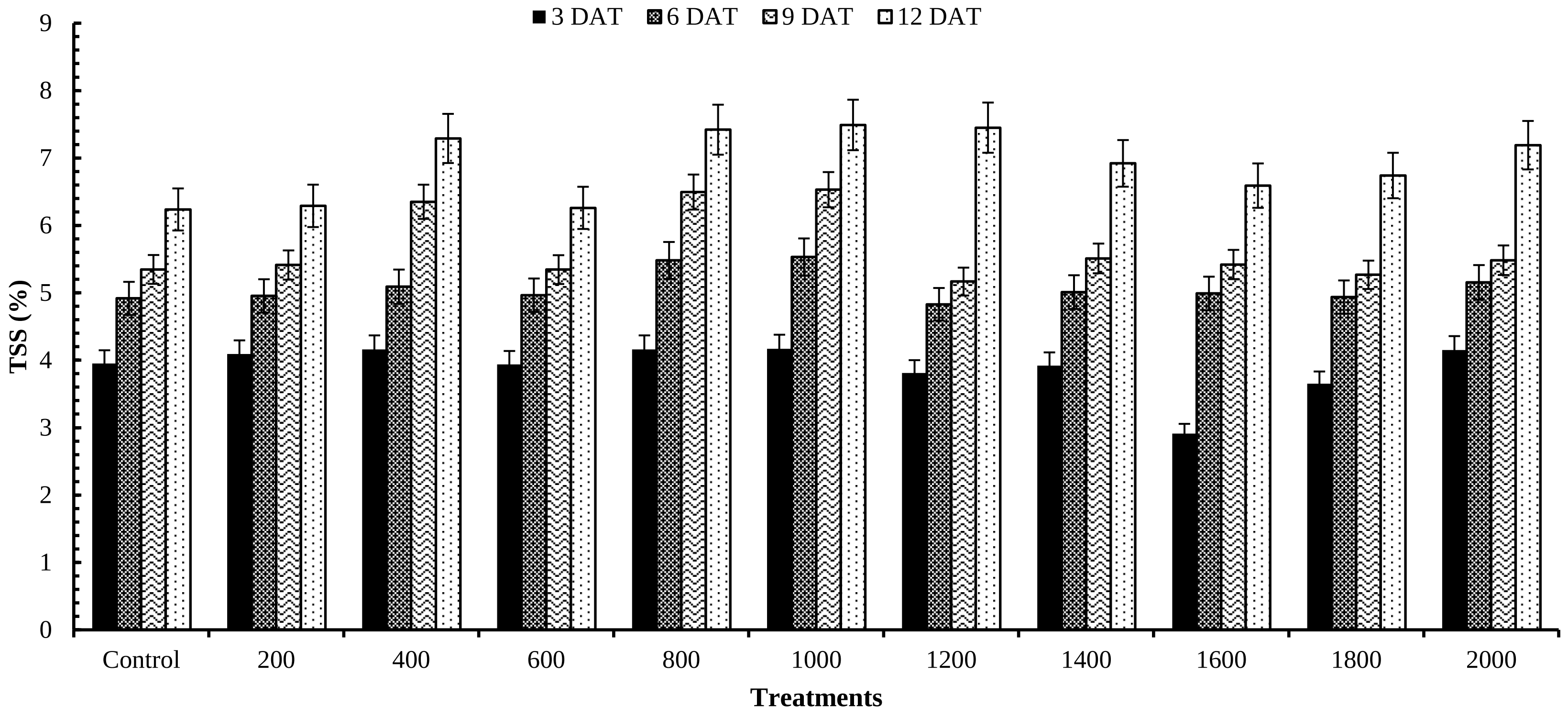
3 DAT
6 DAT
9 DAT
12 DAT
TSS (%)
0
1
2
3
4
5
6
7
8
9
Control
200
400
600
800
1000
1200
1400
1600
1800
2000
Treatments

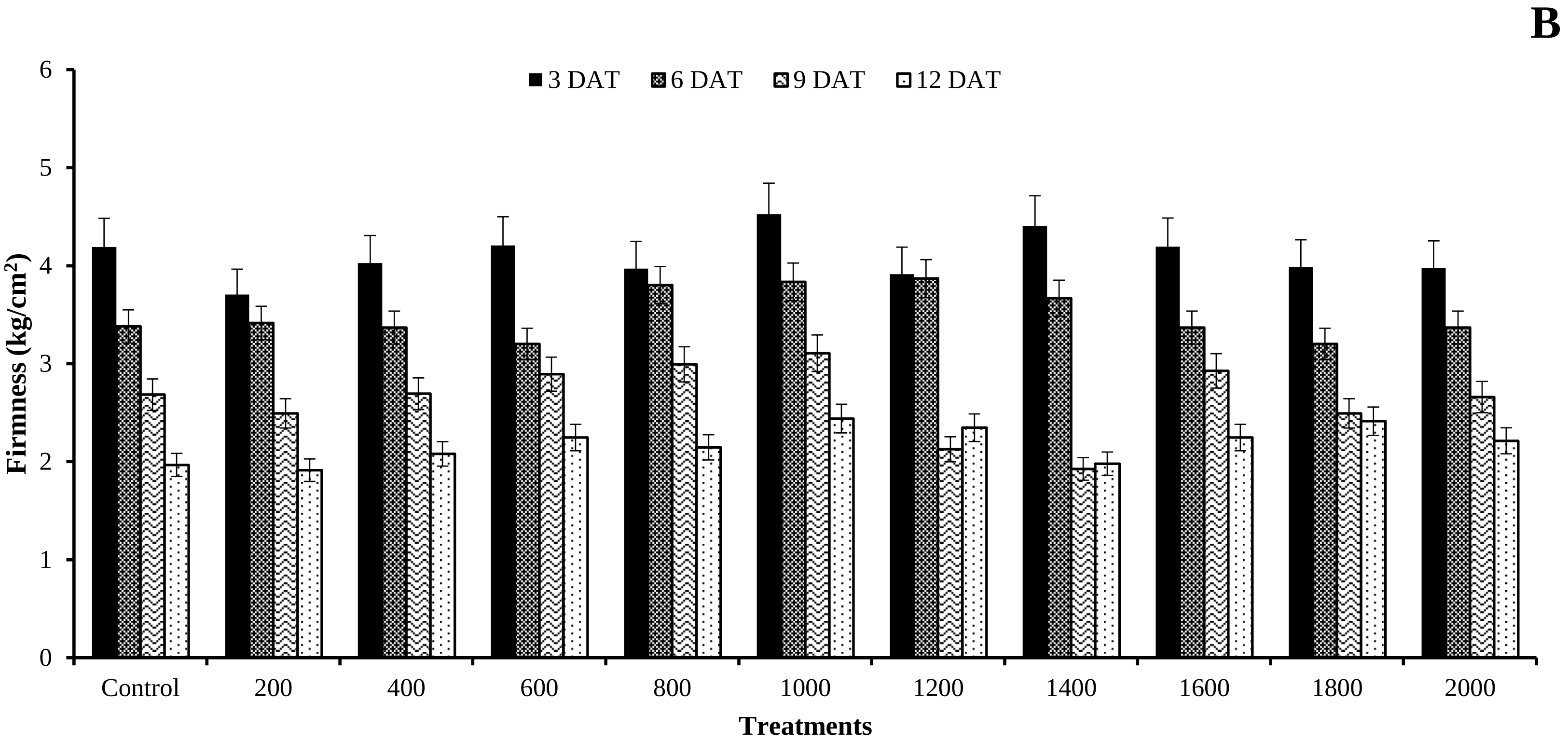

Figure S1. Total Soluble Solid (A) and Firmness (B) of 'Camarosa' strawberry fruits following foliar application of Na_2SeO_4. Control: No application of Na_2SeO_4; 200-2000 treatments: represent the amount of Na_2SeO_4 applied in μM. No statistical differences were observed between the different Na_2SeO_4 concentrations evaluated ($p<0.05$).

Table S1. Effect of the main factors of the variables: Chlorophyll *a*, Chlorophyll *b*, Titratable acidity, Total soluble solid, Hydrogen peroxide, and Malondialdehyde.

Factor	Treatment	Chlorophyll *a*	Chlorophyll *b*	Titratable acidity	Total soluble solid	Hydrogen peroxide	Malondialdehyde
Main plot	3 DAS	0.65	0.27 b	3.43 b	4.18 d	6.34 d	3.05 d
	6 DAS	0.74	0.37 a	3.93 a	5.08 c	8.23 c	4.69 c
	9 DAS	0.78	0.38 a	3.02 c	5.81 b	14.33 b	8.36 b
	12 DAS	0.74	0.37 a	1.98 d	6.81 a	16.60 a	13.29 a
	p-value	0.0618	0.0019	<0.0001	0.0001	<0.0001	<0.0001
Subplot	Control	0.63 b	0.27 b	3.24 a	5.06 b	12.47 a	7.80 a
	NaSe	0.83 a	0.43 a	2.94 b	5.87 a	10.28 b	6.89 b
	p-value	<0.0001	<0.0001	<0.0001	0.0006	<0.0001	0.0458

Table S2. Effect of the main factors of the variables: Total flavonoid, Total phenol, Anthocyanin, Ascorbic Acid, DPPH, SOD, CAT, APX, PAL.

Factor	Treatment	Total flavonoid	Total phenol	Anthocyanin	Ascorbic Acid	DPPH	SOD	CAT	APX	PAL
Main plot	3 DAS	5.77 d	24.24 d	7.38 d	20.13 d	24.92 d	19.95 c	12.06 d	27.87 d	32.58 d
	6 DAS	9.55 c	36.60 c	22.03 c	27.89 c	44.35 c	31.84 b	20.29 c	47.64 c	41.49 c
	9 DAS	20.24 b	65.11 b	59.13 b	50.79 b	49.63 b	53.22 a	47.99 b	74.71 b	72.56 b
	12 DAS	27.64 a	73.01 a	76.75 a	79.14 a	77.49 a	53.96 a	73.35 a	78.56 a	84.05 a
	p-value	<0.0001	<0.0001	<0.0001	<0.0001	<0.0001	<0.0001	<0.0001	<0.0001	<0.0001
Subplot	Control	13.31 b	42.30 b	33.36 b	39.64 b	43.97 b	36.71 b	34.13 b	52.90 b	44.27 b
	NaSe	18.29 a	57.17 a	49.29 a	49.33 a	54.22 a	42.77 a	42.71 a	61.49 a	71.07 a
	p-value	<0.0001	<0.0001	<0.0001	<0.0001	<0.0001	<0.0001	<0.0001	0.0002	<0.0001

Table S3. Effect of the main factors of the variables: ABA, *FaNCED1*, *FaGAMYB*, *FaMYB1*, *FaMYC1*, *FaCHSFaPAL*, and *FaSUT1*.

Factor	Treatment	ABA	*FaNCED1*	*FaGAMYB*	*FaMYB1*	*FaMYC1*	*FaCHS*	*FaPAL*	*FaSUT1*
Main plot	3 DAS	12.36 d	1.17 d	3.47 d	1.82 d	9.43 a	0.62 d	1.38 c	1.03 c
	6 DAS	29.01 c	2.23 c	7.33 b	2.41 c	8.01 b	2.77 c	3.68 b	1.63 b
	9 DAS	97.22 b	3.54 b	9.50 a	5.66 b	6.27 b	6.65 b	3.87 b	3.14 a
	12 DAS	116.46 a	4.59 a	5.31 c	6.17 a	3.30 d	7.40 a	5.73 a	3.35 a

	p-value	<0.0001	<0.0001	<0.0001	<0.0001	<0.0001	<0.0001	<0.0001	<0.0001
**Subplot**	Control	55.80 b	2.68 b	5.96 b	3.61 b	7.21 a	3.46 b	3.18 b	1.98 b
	NaSe	71.72 a	3.08 a	6.85 a	4.42 a	6.29 b	5.26 a	4.15 a	2.60 a
	p-value	<0.0001	0.0005	0.0016	0.0008	0.0090	<0.0001	0.0001	0.0002

Table S4. Primer's information.

**Primer Name**	**Primer Sequence, 5'-3'**	**Length**	**GC, %**	**Tm, °C**	**Type**
FaSUT1 F	TGTTTGTGTTTGGGTTTTGG	20	40	54.3	F
FaSUT1 R	AGTGAGATCAGCAAGGAG	18	50	53.9	R
Faactin F	GGTGTGATGGTTGGGATG	18	55.6	56.1	F
Faactin R	GTAGAAGGTGTGATGCCAAA	20	55.6	56.1	R
FaNCED1 F	ACCCCTAACTTCTTCTTCTTC	21	42.9	57.4	F
FaNCED1 R	GGTTTTGGGGAGTGGATG	18	55.6	56.1	R
FaMYB1 F	GGAAGGACAGATAACGAA	18	44.4	51.6	F
FaMYB1 R	TTTGGACGAAGAGTAGTG	18	44.4	51.6	R
FaPAL F	GGAGTATTTGGCAAGGGA	18	50	53.9	F
FaPAL R	GGAGTAATGTTGTGGTTGAG	20	50	53.9	R
FaMYC1 F	GTGTCCTTTCTTCCCTTT	18	44.4	51.6	F
FaMYC1 R	TCCTCCTTTCTTCCAACTC	19	47.4	55.2	R
FaCHS F	CTCACATTTCACCTCCTC	18	50	53.9	F
FaCHS R	TCGTGGCTTCTAACTTCT	18	44.4	51.6	R
FaGAMYB F	AGATGGGAAACAAATGGG	18	44.4	51.6	F
FaGAMYB R	AGGAGGATAAAGTGGTAAG	19	42.1	53.0	R
